# Blood urea nitrogen to albumin ratio as predictor of mortality among acute pancreatitis patients in ICU: A retrospective cohort study

**DOI:** 10.1371/journal.pone.0323321

**Published:** 2025-05-15

**Authors:** Wei Li, Ning Li, Yujia Zhan, Jun Gu

**Affiliations:** Department of Cardiovascular Surgery, West China Hospital, Sichuan University, Chengdu, China; Osaka University of Pharmaceutical Sciences, JAPAN

## Abstract

**Objective:**

Blood urea nitrogen to albumin ratio (BAR) has served as a predictive marker for patients in the Intensive Care Unit (ICU), and has been studied in patients with sepsis, post-cardiac surgery, severe COVID-19, and acute exacerbation of chronic obstructive pulmonary disease (AECOPD). This objective indicator has demonstrated capability in prognostic prediction.However, research on the prognostic value of BAR in acute pancreatitis (AP) patients are scarce,the goal was to explore the relationship between BAR and total mortality in AP admitted to ICU.

**Methods:**

A Retrospective analysis was performed utilizing the Medical Information Market for Intensive Care (MIMIC IV) database. Patients with AP admitted to ICU were included and grouped based on BAR. Univariate and multivariate Cox regression analysis were utilized to explore the relationship between BAR and total mortality. The area under the curve (AUC) of the receiver operating characteristic (ROC) curve was applied to assess the predictive value of BAR. Cumulative hazard risk accumulation curve verified BAR’s predictive capability for short- and long-term mortality. Heterogeneity between different subgroups was excluded by subgroup analysis.

**Results:**

Total 514 AP patients were divided into high-BAR (BAR ≥ 7.62) and low-BAR group (BAR < 7.62). The duration of ICU stay was significantly extended in the high BAR group. In the Cox proportional hazard model, whether adjusting for confounding factors or not, the high BAR was an independent risk factor for total mortality. AUC for BAR was 0.78 (95% C1: 0.72–0.84) at 28 days and 0.70 (95%: Cl: 0.64–0.75) at 360 days.

**Conclusion:**

BAR is an objective and independent predictor of both short- and long-term total mortality in AP patients. A prompt, efficient, and uncomplicated assessment of the severity and prognosis, which facilitates ICU doctors to develop treatment plans for poor patient outcomes.

## 1. Introduction

Acute pancreatitis (AP) is a prevalent gastrointestinal disorder observed in intensive care unit (ICU), with over 300,000 annual visits to the emergency department in the United States. Its incidence is estimated to be 110–140 per 100,000 people, and the hospitalization cost per hospitalization is close to $ 7,000. The annual cost of hospitalization has exceeded $2.5 billion [1–3]. AP is marked by acinar cell injury affecting exocrine function. The inappropriate activation of trypsinogen in acinar cells triggers the activation of other digestive enzymes, kallikrein-kinin system and complement cascades. This leads to self-digestion of pancreatic tissue. Activated trypsin and inflammatory factors continue to escalate, leading to the manifestation of edema, hemorrhage, and necrosis in pancreatic tissue. The involvement of additional organs and tissues result in organ dysfunction [4]. Studies indicate that around 80% of patients develop mild to moderate symptoms, while 20% experience severe pancreatitis with a 20% mortality rate. It can be quite challenging to anticipate disease progression at an early stage and the management of complications not only increases patient suffering but also consumes more medical resources [5,6]. Thus, effective assessment of AP severity is essential in early phase and timely intervention measures are of critical importance in reducing mortality in AP.

Blood urea nitrogen (BUN) and serum albumin (ALB) are easily accessible and provide objective results in routine clinical blood tests. Prior research has established the BUN to ALB ratio (BAR) as a significant forecast index in sepsis, post-cardiac surgery, severe COVID-19,and acute exacerbation of chronic obstructive pulmonary disease (AECOPD) [7–11]. The predictive potential of BAR for disease severity can assist in clinical treatment decisions and patient management. No studies have examined the role of BAR in predicting the prognosis of adult AP patients in ICU. In order to make up for this gap, we analysed the admission and follow-up data from MIMIC-IV database to investigate the correlation between BAR and total mortality in AP patients.

## 2. Methods

### 2.1. Database

Primary dataset for our study was sourced from MIMIC-IV database (version 2.2). Including medical records of adults with ICU at Beth Israel Deaconess Medical Center in Boston, Massachusetts, from 2008 to 2019 [12]. All patient info were anonymized records and can be accessed publicly after authorization. Database authorization is granted (No: 10323541),upon completion of the online training,thus exempting ethical approval and informed consent requirements.

### 2.2. Data collection

**Fig 1:**
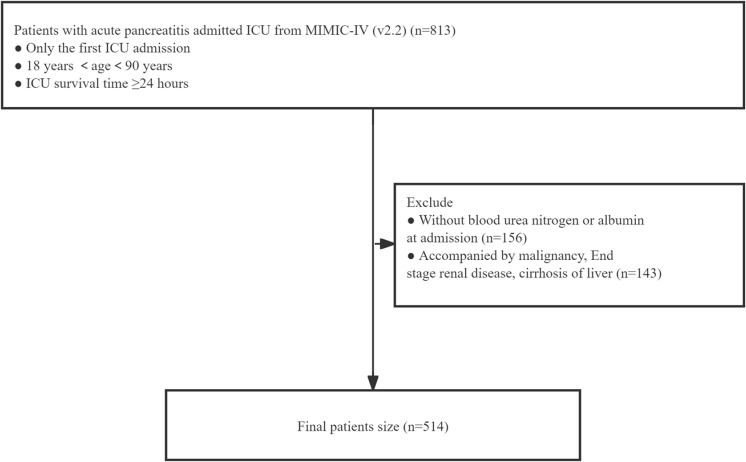
Data extraction and exclusion protocol.

We used the Structured Query Language (SQL) on the Navicat (16.2.11) software to extract variable information. In order to reduce treatment interference and ensure the homogeneity of the baseline data, BAR was used as the main research study variable to extract the first vital signs, initial laboratory test data within 24 hours of ICU admission, demographic information, clinical scores, treatment plan, complications, and prognosis of patients after ICU admission. Data from a total of 813 AP sufferers were collected from 2012 to 2019. Inclusion criteria: (1) ages between 18–90 years; (2) ICU admission for treatment; (3) survival time exceeding 24 hours since admission to ICU (The first ICU record data was selected for a sufferer with multiple admissions). The following criteria are excluded: (1) Missing BUN and ALB data at ICU admission; (2) Patients with end-stage renal disease, cirrhosis, or malignant tumors. Eventually, 514 patients participated in our research (Fig 1).

### 2.3. Primary outcomes

The primary endpoint was total mortality within 28, 60, 90, and 360 days following patient transfer to ICU. The secondary outcome was the length of ICU stay, ICU mortality, and total length of hospitalization.

### 2.4. Management data

To mitigate bias, indicators with a missing value exceeding 20% were excluded (e.g., height, PCO2, PO2, TCO2, FCa2+). For variables with data missing no more than 20% the multiple imputation method supplements it (e.g., weight, NBPm, T, WBC, RBC, Plt, HB, RDW, HCT, K + , pH, Lac, PT, APTT, INR, Tbil, ALT, AST).

### 2.5. Statistical analysis

Our study utilized t-tests and Mann-Whitney U tests for continuous variables, presenting non-normally distributed baseline characteristic as median (interquartile range, IQR). Chi-square and Fisher’s exact tests were employed for categorical variable comparisons, displayed as numbers (percentages). The Receiver Operating Characteristic (ROC) curve was generated to establish the BAR threshold for patients who died within 28 days of ICU admission. With the cutoff value, patients were divided into high-BAR (BAR ≥ 7.62) and low-BAR groups (BAR < 7.62), analysis of clinical characteristics and endpoint events. To clarify independent predictors of in-hospital mortality at 28, 60, 90, and 360 days, we performed both univariate and multivariate Cox regression analyses. HR with 95% confidence interval (CI) is given. The cumulative hazard risk accumulation curve was utilized to assess survival rate variations among the BAR groups. The area under the curve (AUC) of the ROC curve was utilized to evaluate and compare the predictive efficacy of BAR and other scoring systems (e.g., LAR, CAR, RAR, SOFA, and OASIS) on all-cause death in AP patients. SPSS Statistics software Version 29.0.0 (International Business Machines Corporation, Armonk, NY, USA) was utilized for investigation.

## 3. Results

### 3.1. Baseline variables

Table 1 presents baseline characteristics of AP within 28 days following ICU admission, categorized by survivor and expired. Ultimately,514 inpatients were involved in this study, including 218 (42%) female and 296 (58%) male. Median age was 58 years(46.00–70.75). The mortality rate within 28 days in the ICU was 7.0%, with the deceased group being older [73.50 (63.00–81.75) vs 56.00 (45.00–68.00), P < 0.001], lower body temperature [36.60 (36.44–36.94) vs 36.94 (36.56–37.44), P < 0.001], higher SOFA score [9.00 (5.75–12.00) vs 4.00 (2.00–7.00), P < 0.001], and higher OASIS score [41.00 (35.00–46.00) vs 31.00 (26.00–38.00), P < 0.001]. The incidence of AKI and sepsis in the expired group was 86% and 89%, For therapeutic interventions, ventilators were used in 33% of the non-survival group and CRRT in 15%. On laboratory indices, the BAR in the expired group was significantly elevated [14.08 (8.74–20.12) vs. 6.15 (3.58–10.29), P < 0.001]. Comparison of survival and mortality groups revealed RDW, [14.40 (13.50–15.58) VS 15.20 (14.35–16.55), P = 0.003], Cr, [1.00 (0.70–1.60) VS 1.50 (1.08–2.25), P < 0.001], BUN, [18.00 (11.00–29.00) VS 32.50 (19.75–43.75), P < 0.001], sodium, [138.00 (135.00–141.00) VS 140.00 (137.00–145.25), P = 0.007], chloride, [104.00 (100.00–109.00) VS 109.50 (104.00–112.25),P = 0.001], APTT, [30.10 (26.83–34.41) VS 34.30 (28.40–41.18), P = 0.021], lac, [1.80 (1.20–2.63) VS 2.05 (1.58–3.93), P = 0.025], and anion gap, [15.00 (13.00–18.00) VS 18.00 (13.00–21.25), P = 0.029]. The survival values are lower than mortality group (P < 0.05) which assumes statistical significance.

**Table 1:** Baseline of variable among survivor and death within 28 days.

Variable	Overall, N = 514^1^	Survivor, N = 478^1^	Expired, N = 36^1^	p-value^2^
**Demographics**				
Age, year,Median (IQR)	58.00 (46.00–70.75)	56.00 (45.00–68.00)	73.50 (63.00–81.75)	<0.001
Gender, n (%)				0.10
Male	296 (58)	280 (59)	16 (44)	
Female	218 (42)	198 (41)	20 (56)	
Weight, kg,Median (IQR)	84.70 (70.78–101.40)	85.14 (71.11–101.84)	75.85 (67.93–91.83)	0.15
**Vital Signs**				
HR,bpm, Median (IQR)	100.00 (84.25–116.00)	100.00 (85.00–115.75)	101.00 (82.75–118.75)	0.68
RR, bpm,Median (IQR)	20.00 (17.00–25.00)	20.50 (17.00–25.00)	20.00 (18.00–25.25)	0.92
SPO2, %,Median (IQR)	96.00 (94.00–99.00)	96.00 (94.00–99.00)	98.00 (95.00–99.00)	0.12
NBPm, mmHg,Median (IQR)	86.00 (73.00–99.00)	87.00 (73.25–99.00)	81.85 (69.50–97.25)	0.34
T,°C, Median (IQR)	36.89 (36.56–37.39)	36.94 (36.56–37.44)	36.60 (36.44–36.94)	<0.001
**Laboratory data**				
WBC, k/uL,Median (IQR)	12.80 (8.90–18.20)	12.60 (8.90–18.10)	14.75 (9.18–19.28)	0.28
RBC,m/uL, Median (IQR)	3.70 (3.22–4.22)	3.72 (3.23–4.22)	3.55 (3.17–4.49)	0.93
Plt, k/uL,Median (IQR)	197.00 (138.25–277.75)	198.00 (140.25–280.00)	168.00 (125.50–226.50)	0.17
Hb, g/dL,Median (IQR)	11.30 (9.70–13.00)	11.30 (9.73–13.00)	11.15 (9.70–13.20)	0.58
RDW, %,Median (IQR)	14.50 (13.60–15.60)	14.40 (13.50–15.58)	15.20 (14.35–16.55)	0.003
HCT, %,Median (IQR)	34.05 (29.50–38.95)	34.05 (29.50–38.78)	34.15 (29.83–39.03)	0.93
Alb,g/dL, Median (IQR)	2.90 (2.50–3.30)	3.00 (2.50–3.40)	2.45 (2.00–2.83)	<0.001
TBil, Median (IQR)	1.00 (0.60–2.50)	1.00 (0.60–2.50)	1.10 (0.70–2.20)	0.42
ALT, IU/L,Median (IQR)	53.50 (24.00–141.00)	53.50 (24.00–139.50)	52.30 (24.50–143.75)	0.78
AST,IU/L,Median (IQR)	69.50 (34.00–168.25)	69.00 (33.00–168.25)	85.00 (40.75–154.75)	0.34
BUN, mg/dL,Median (IQR)	18.00 (11.00–31.75)	18.00 (11.00–29.00)	32.50 (19.75–43.75)	<0.001
Cr, mg/dL,Median (IQR)	1.00 (0.70–1.60)	1.00 (0.70–1.60)	1.50 (1.08–2.25)	<0.001
BAR, Median (IQR)	6.38 (3.68–10.98)	6.15 (3.58–10.29)	14.08 (8.74–20.12)	<0.001
Na, mEq/L,Median (IQR)	138.00 (135.00–141.00)	138.00 (135.00–141.00)	140.00 (137.00–145.25)	0.007
K,mEq/L, Median (IQR)	4.00 (3.60–4.48)	4.00 (3.60–4.40)	4.25 (3.88–4.85)	0.053
TCa2, mg/dL,Median (IQR)	7.90 (7.30–8.40)	7.90 (7.30–8.40)	7.80 (7.00–8.23)	0.30
Cl,mEq/L, Median (IQR)	104.00 (101.00–109.00)	104.00 (100.00–109.00)	109.50 (104.00–112.25)	0.001
Glu, mg/dL,Median (IQR)	131.00 (105.00–175.75)	130.00 (105.00–174.00)	142.00 (115.00–181.50)	0.17
PH, Median (IQR)	7.36 (7.29–7.41)	7.36 (7.29–7.41)	7.36 (7.27–7.39)	0.33
Lac, mmol/L,Median (IQR)	1.81 (1.30–2.70)	1.80 (1.20–2.63)	2.05 (1.58–3.93)	0.025
AG, mEq/L,Median (IQR)	15.00 (13.00–18.00)	15.00 (13.00–18.00)	18.00 (13.00–21.25)	0.029
PT, s,Median (IQR)	14.20 (12.82–16.08)	14.10 (12.82–15.92)	15.15 (12.85–17.68)	0.13
APTT, s, Median (IQR)	30.20 (26.93–35.00)	30.10 (26.83–34.41)	34.30 (28.40–41.18)	0.021
INR, Median (IQR)	1.30 (1.10–1.50)	1.30 (1.10–1.50)	1.35 (1.20–1.63)	0.080
**Scores**				
SOFA, Median (IQR)	5.00 (2.00–8.00)	4.00 (2.00–7.00)	9.00 (5.75–12.00)	<0.001
OASIS, Median (IQR)	32.00 (26.00–39.00)	31.00 (26.00–38.00)	41.00 (35.00–46.00)	<0.001
**Comorbidities**				
AKI, n (%)				0.002
NO	195 (38)	190 (40)	5 (14)	
YES	319 (62)	288 (60)	31 (86)	
Sepsis, n (%)				<0.001
NO	189 (37)	185 (39)	4 (11)	
YES	325 (63)	293 (61)	32 (89)	
HT, n (%)				0.62
NO	251 (49)	232 (49)	19 (53)	
YES	263 (51)	246 (51)	17 (47)	
DM, n (%)				0.50
NO	360 (70)	333 (70)	27 (75)	
YES	154 (30)	145 (30)	9 (25)	
HF, n (%)				0.007
NO	437 (85)	412 (86)	25 (69)	
YES	77 (15)	66 (14)	11 (31)	
CKD, n (%)				0.047
NO	457 (89)	429 (90)	28 (78)	
YES	57 (11)	49 (10)	8 (22)	
**Clinical treatments**				
MV, n (%)				<0.001
NO	311 (61)	299 (63)	12 (33)	
YES	203 (39)	179 (37)	24 (67)	
CRRT, n (%)				<0.001
NO	466 (91)	445 (93)	21 (58)	
YES	48 (9.3)	33 (6.9)	15 (42)	
Epinephrine, n (%)				<0.001
NO	496 (96)	468 (98)	28 (78)	
YES	18 (3.5)	10 (2.1)	8 (22)	
Norepinephrine, n (%)				<0.001
NO	383 (75)	372 (78)	11 (31)	
YES	131 (25)	106 (22)	25 (69)	
Vasopressin, n (%)				<0.001
NO	449 (87)	431 (90)	18 (50)	
YES	65 (13)	47 (9.8)	18 (50)	
**Outcomes**				
Los_Hosp,day, Median (IQR)	13.04 (7.39–23.87)	13.67 (7.52–24.82)	9.72 (3.80–15.92)	0.004
Los_Icu,day, Median (IQR)	3.13 (1.70–8.30)	3.09 (1.67–8.21)	5.87 (2.05–10.18)	0.10
Mortality, n (%)				<0.001
NO	389 (76)	389 (81)	0 (0)	
YES	125 (24)	89 (19)	36 (100)	
In hospital mortality, n (%)				<0.001
NO	474 (92)	470 (98)	4 (11)	
YES	40 (7.8)	8 (1.7)	32 (89)	
ICU mortality, n (%)				<0.001
NO	487 (95)	475 (99)	12 (33)	
YES	27 (5.3)	3 (0.6)	24 (67)	

^1^Median (IQR) or Frequency (%).

^2^Chi-squared test; Wilcoxon rank sum test; Fisher’s exact test.

HR, heart rate; RR, respiratory rate; SPO2, Pulse Oxygen Saturation; NBPm, mean non-invasive blood pressure; T,temperature; WBC, white blood cell; RBC,red blood cell; Plt, platelet; Hb, hemoglobin; RDW, Red Distribution Width; Hct, hematocrit; Alb, albumin; TBil, total bilirubin; ALT, alanine aminotransferase; AST, aspartate aminotransferase; BUN, blood urea nitrogen; Cr, creatinine; Na, serum sodium; K, serum potassium; TCa, serum total calcium; Cl, serum chloride; Glu, glucose; Lac, lactate; AG, anion gap; PT, prothrombin time; APTT, activated partialthromboplastin time; INR, international normalized ratio; BAR, Serum urea nitrogen to albumin ratio; MV, mechanical ventilation; CRRT, continuous renal replacement therapy; AKI, acute kidney injury; HT, Hypertension; DM, diabetes; HF, heart failure; CKD, chronic kidney disease; AF, atrial fibrillation; LOS_ICU, length of ICU stay; LOS_ Hosp, length of hospital stay.

### 3.2 Relationship between BAR and clinical variables

**Fig 2:**
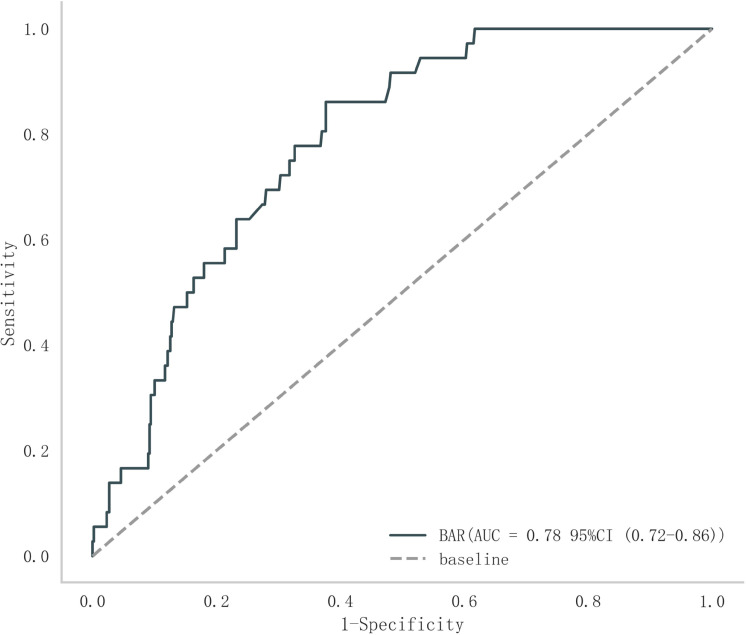
BAR cut-off value for maximizing the risk ratio of death within 28 days.

The clinical characteristics and end-point events were analyzed. The area under the ROC curve (AUC) within 28 days was 0.78 (95% CI: 0.72–0.86), the sensitivity was 0.86 (0.73–1.00), and the specificity was 0.62 (0.44–0.82). The BAR cut-off value that maximizes the risk ratio is 7.62 (Fig 2).

Participants were categorized into high-BAR containing 303 patients (BAR ≥ 7.62) and low-BAR containing 211 patients (BAR < 7.62) based on cut-off value. Presentation in Table 2, the two groups exhibit a statistical significance in age, NBPm, T, WBC, PLT, RDW, Crea, Na, K, Cl, pH, LAC, AG, PT, SOFA, OASIS, and SAPSII (P < 0.05). No notable differences were found in remaining covariates (P > 0.05).

**Table 2:** Baseline characteristics stratifed by BAR.

Variable	Overall, N = 514^1^	BAR ＜ 7.62, N = 303^1^	BAR ≥ 7.62, N = 211^1^	p-value^2^
**Demographics**				
Age, year,Median (IQR)	58.00 (46.00–70.75)	53.00 (41.00–65.00)	64.00 (54.00–76.00)	<0.001
Gender, n (%)				0.93
Male	296 (58)	174 (57)	122 (58)	
Female	218 (42)	129 (43)	89 (42)	
Weight, kg,Median (IQR)	84.70 (70.78–101.40)	83.30 (69.25–101.15)	86.40 (72.49–103.70)	0.14
**Vital Signs**				
HR,bpm, Median (IQR)	100.00 (84.25–116.00)	101.00 (84.00–116.50)	99.00 (86.00–114.50)	0.87
RR, bpm,Median (IQR)	20.00 (17.00–25.00)	20.00 (17.00–24.50)	21.00 (16.00–25.50)	0.53
SPO2, %,Median (IQR)	96.00 (94.00–99.00)	96.00 (94.00–99.00)	96.00 (94.00–98.00)	0.21
NBPm, mmHg,Median (IQR)	86.00 (73.00–99.00)	90.00 (78.00–102.00)	81.00 (69.00–93.00)	<0.001
T,°C, Median (IQR)	36.89 (36.56–37.39)	37.00 (36.67–37.59)	36.72 (36.44–37.14)	<0.001
**Laboratory data**				
WBC, k/uL, Median (IQR)	12.80 (8.90–18.20)	12.00 (8.35–16.30)	14.30 (9.95–19.95)	<0.001
RBC, m/uL, Median (IQR)	3.70 (3.22–4.22)	3.73 (3.27–4.22)	3.64 (3.13–4.32)	0.52
Plt, k/uL, Median (IQR)	197.00 (138.25–277.75)	205.00 (148.50–286.50)	190.00 (129.50–250.00)	0.033
Hb, g/dL, Median (IQR)	11.30 (9.70–13.00)	11.40 (9.90–12.90)	11.20 (9.50–13.30)	0.49
RDW, %, Median (IQR)	14.50 (13.60–15.60)	14.30 (13.45–15.40)	14.70 (13.85–15.90)	0.002
HCT, %, Median (IQR)	34.05 (29.50–38.95)	34.00 (29.95–38.05)	34.10 (28.75–40.00)	0.94
Alb, g/dL, Median (IQR)	2.90 (2.50–3.30)	3.10 (2.70–3.50)	2.70 (2.30–3.10)	<0.001
TBil, Median (IQR)	1.00 (0.60–2.50)	0.90 (0.60–2.20)	1.10 (0.55–2.90)	0.41
ALT, IU/L, Median (IQR)	53.50 (24.00–141.00)	53.00 (22.00–144.00)	55.00 (25.00–133.50)	0.61
AST, IU/L,Median (IQR)	69.50 (34.00–168.25)	61.00 (31.00–151.50)	78.00 (40.50–179.50)	0.047
BUN, mg/dL, Median (IQR)	18.00 (11.00–31.75)	12.00 (8.00–17.00)	35.00 (26.00–52.00)	<0.001
Cr, mg/dL, Median (IQR)	1.00 (0.70–1.60)	0.80 (0.60–1.00)	1.80 (1.20–3.05)	<0.001
Na, mEq/L, Median (IQR)	138.00 (135.00–141.00)	138.00 (135.00–140.00)	140.00 (135.00–143.00)	0.005
K, mEq/L, Median (IQR)	4.00 (3.60–4.48)	3.90 (3.50–4.30)	4.20 (3.70–4.80)	<0.001
TCa2,mg/dL, Median (IQR)	7.90 (7.30–8.40)	8.00 (7.40–8.50)	7.70 (7.00–8.30)	<0.001
Cl, mEq/L, Median (IQR)	104.00 (101.00–109.00)	104.00 (100.00–108.00)	106.00 (101.00–111.00)	<0.001
Glu, mg/dL, Median	131.00 (105.00–175.75)	125.00 (105.00–164.50)	141.00 (107.50–188.00)	0.005
PH, Median (IQR)	7.36 (7.29–7.41)	7.38 (7.33–7.42)	7.33 (7.25–7.39)	<0.001
Lac, mmol/L, Median (IQR)	1.81 (1.30–2.70)	1.71 (1.20–2.50)	2.00 (1.30–3.00)	0.003
AG, mEq/L, Median (IQR)	15.00 (13.00–18.00)	14.00 (12.00–16.50)	17.00 (14.00–20.00)	<0.001
PT, s, Median (IQR)	14.20 (12.82–16.08)	13.80 (12.70–15.70)	14.70 (13.00–17.45)	<0.001
APTT, s, Median (IQR)	30.20 (26.93–35.00)	29.70 (26.70–34.30)	31.30 (27.20–37.10)	0.052
INR, Median (IQR)	1.30 (1.10–1.50)	1.20 (1.10–1.40)	1.30 (1.20–1.60)	<0.001
**Scores**				
SOFA, Median (IQR)	5.00 (2.00–8.00)	3.00 (2.00–6.00)	7.00 (4.00–10.00)	<0.001
OASIS, Median (IQR)	32.00 (26.00–39.00)	30.00 (25.00–35.00)	36.00 (29.00–43.00)	<0.001
**Comorbidities**				
AKI, n (%)				<0.001
NO	195 (38)	150 (50)	45 (21)	
YES	319 (62)	153 (50)	166 (79)	
Sepsis, n (%)				<0.001
NO	189 (37)	146 (48)	43 (20)	
YES	325 (63)	157 (52)	168 (80)	
HT, n (%)				0.28
NO	251 (49)	142 (47)	109 (52)	
YES	263 (51)	161 (53)	102 (48)	
DM, n (%)				0.056
NO	360 (70)	222 (73)	138 (65)	
YES	154 (30)	81 (27)	73 (35)	
HF, n (%)				0.002
NO	437 (85)	270 (89)	167 (79)	
YES	77 (15)	33 (11)	44 (21)	
CKD, n (%)				<0.001
NO	457 (89)	290 (96)	167 (79)	
YES	57 (11)	13 (4.3)	44 (21)	
**Clinical treatments**				
MV, n (%)				<0.001
NO	311 (61)	206 (68)	105 (50)	
YES	203 (39)	97 (32)	106 (50)	
CRRT, n (%)				<0.001
NO	466 (91)	293 (97)	173 (82)	
YES	48 (9.3)	10 (3.3)	38 (18)	
Epinephrine, n (%)				0.078
NO	496 (96)	296 (98)	200 (95)	
YES	18 (3.5)	7 (2.3)	11 (5.2)	
Norepinephrine, n (%)				<0.001
NO	383 (75)	256 (84)	127 (60)	
YES	131 (25)	47 (16)	84 (40)	
Vasopressin, n (%)				<0.001
NO	449 (87)	287 (95)	162 (77)	
YES	65 (13)	16 (5.3)	49 (23)	
**Outcomes**				
Mortality, n (%)				<0.001
NO	389 (76)	253 (83)	136 (64)	
YES	125 (24)	50 (17)	75 (36)	
In hospital mortality, n (%)				<0.001
NO	474 (92)	296 (98)	178 (84)	
YES	40 (7.8)	7 (2.3)	33 (16)	
ICU mortality, n (%)				<0.001
NO	487 (95)	299 (99)	188 (89)	
YES	27 (5.3)	4 (1.3)	23 (11)	
Death_within_ICU_28days, n (%)				<0.001
NO	478 (93)	298 (98)	180 (85)	
YES	36 (7.0)	5 (1.7)	31 (15)	
Death_within_ICU_60days, n (%)				<0.001
NO	459 (89)	291 (96)	168 (80)	
YES	55 (11)	12 (4.0)	43 (20)	
Death_within_ICU_90days, n (%)				<0.001
NO	447 (87)	288 (95)	159 (75)	
YES	67 (13)	15 (5.0)	52 (25)	
Death_within_ICU_360days, n (%)				<0.001
NO	424 (82)	275 (91)	149 (71)	
YES	90 (18)	28 (9.2)	62 (29)	
Los_Hosp,day, Median (IQR)	13.04 (7.39–23.87)	10.74 (6.79–19.81)	16.65 (9.68–28.36)	<0.001
Los_ICU,day, Median (IQR)	3.13 (1.70–8.30)	2.93 (1.53–5.87)	4.38 (1.87–12.58)	<0.001

^1^Median (IQR) or Frequency (%).

^2^Chi-squared test;Wilcoxon rank sum test; Fisher’s exact test.

Death_within_ICU_28, 60, 90, 360 days, the difference between ICU intime and dead time is 28 days, 60 days, 90 days, 360 days.

### 3.3. Univariate and Multivariate Cox regression analysis in different models

Univariate Cox analysis was performed on the risk factors associated with mortality within 28 days of ICU admission, aiming to elucidate the potential relationship between BAR and mortality. The results indicated that BAR was identified as a significant independent risk factor, and BAR ≥ 7.62 group exhibited an elevated mortality rate (P < 0.05) (S1 Table). Prognostic indicators at different time periods (28, 60, 90, and 360 days) were analysed by the Cox proportional hazards model. The results indicated that in crude model 0, elevated BAR (>7.62) showed positive correlation with a higher risk of mortality over different time periods [28 days (HR =

9.5 95% CI: 3.69–24.43, P = 0), 60 days (HR = 5.67, 95% CI: 2.99–10.76, P = 0), 90 days (HR = 5.59, 95% CI: 3.15–9.94, P = 0), 360 days (HR = 3.7, 95% CI: 2.37–5.78, P = 0)]. Elimination of confounding factors across different models, in the multivariable model 1 (age, gender,), patients in BAR ≥ 7.62 group had a significantly higher risk of death [28days (HR = 6.83, 95% CI: 2.62–17.82, P = 0), 60days (HR = 4.9, 95% CI: 2.08–7.68, P = 0), 90days (HR = 3.97, 95% CI: 2.21–7.13, P = 0), 360days (HR = 2.7, 95% CI: 1.7–4.26, P = 0)]. Multivariate models 2–6 included additional possible confounders (temperature, complications, clinical score, auxiliary examination, and treatment intervention). Patients in the BAR ≥ 7.62 group were all at higher risk of death (Table 3).

**Table 3:** Multivariate Cox regression analysis for mortality in BAR among different models.

Variable	Model0	Model1	Model2	Model3	Model4	Model5	Model6
**HR (95% CI)**	**HR (95% CI)**	**HR (95% CI)**	**HR (95% CI)**	**HR (95% CI)**	**HR (95% CI)**	**HR (95% CI)**
BAR ＜ 7.62	Reference	Reference	Reference	Reference	Reference	Reference	Reference
28days							
BAR ≥ 7.62	9.5 (3.69,24.43)	6.83(2.62,17.82)	6.32(2.40,16.63)	4.88(1.82,13.09)	3.96(1.44,10.94)	4.40(1.48,13.02)	4.30(1.42,13.05)
P	0	0	0	0	0.01	0.01	0.01
60days							
BAR ≥ 7.62	5.67(2.99,10.76)	4.0(2.08,7.68)	3.67(1.9,7.11)	2.89(1.47,5.69)	2.26(1.12,4.57	2.59(1.22,5.52)	2.48(1.16,5.34)
P	0	0	0	0	0.02	0.01	0.02
90days							
BAR ≥ 7.62	5.59(3.15,9.94)	3.97(2.21,7.13)	3.64(2.01,6.58)	3.04 (1.65,5.6)	2.46 (1.30,4.64)	2.86(1.45,5.64)	2.75(1.39,5.43)
P	0	0	0	0	0.01	0	0
360days							
BAR ≥ 7.62	3.7(2.37,5.78)	2.7(1.7,4.26)	2.44 (1.53,3.88)	2.14(1.32,3.49)	1.74(1.04,2.91)	2.01(1.16,3.47)	1.95 (1.12,3.38)
P	0	0	0	0	0.03	0.01	0.02

Model 0, Non-adjustment;

Model 1, Model 0 + age, gender; Model 2, Model 1 + temperature;

Model 3, Model 2 + complications; Model 4, Model 3 + clinical scores;

Model 5, Model 4 + auxiliary examination; Model 6, Model 5 + treatment intervention.

### 3.4. ROC and Cumulative hazard risk accumulation curves in different intervals

**Fig 3:**
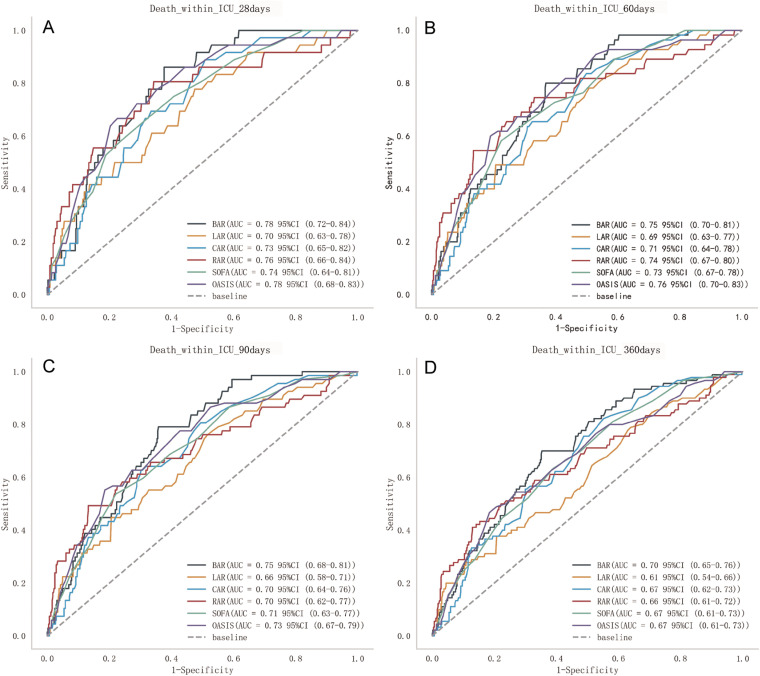
ROC curves analysis for AP patients in different scores and intervals ROC curves analysis different indicators and scores for post ICU admission mortality of AP patients at (A)28 days, (B) 60 days, (C) 90 days, (D) 360 days.

**Fig 4:**
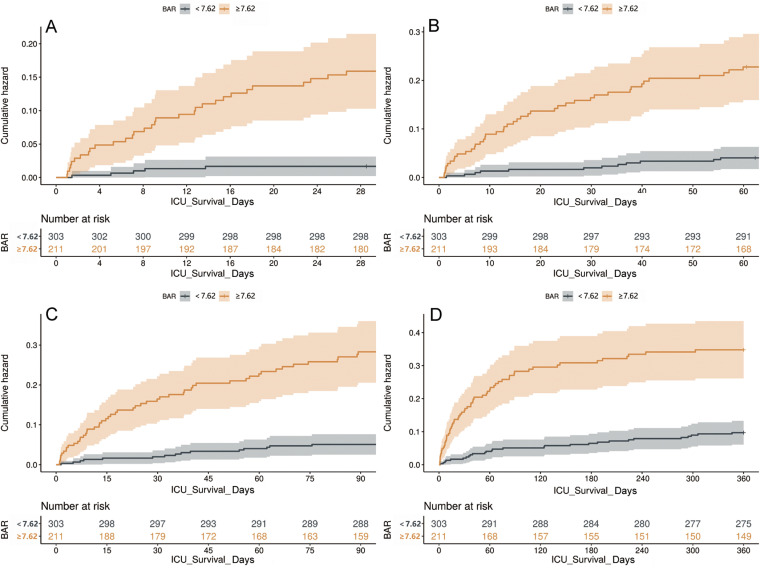
Cumulative hazard risk accumulation curves at different time intervals AP patients admitted to ICU were grouped by BAR < 7.62 and BAR ≥ 7.62, Cumulative hazard risk accumulation curves are displayed for the following intervals: (A) 28 days, (B) 60 days, (C) 90 days, (D) 360 days.

ROC for total mortality in AP patients were analysed and compared with BAR, RAR, CAR, LAR, SOFA and OASIS scores (Fig 3). The comprehensive analysis indicates that value for BAR surpasses that of RAR, CAR, LAR, SOFA, and OASIS scores. Death within ICU 28 days, BAR (AUC = 0.78 95% CI: 0.72–0.84) was better than LAR (AUC = 0.70 95% CI: 0.63–0.78), CAR (AUC = 0.73 95% CI: 0.65–0.82), RAR(AUC = 0.76 95% CI: 0.66–0.84), SOFA (AUC = 0.74 95% CI: 0.64–0.81), and OASIS(AUC = 0.78 95%CI: 0.68–0.83). Death within ICU 360 days, BAR (AUC = 0. 70 95% CI: 0. 65–0. 76) was also higher than LAR (AUC = 0. 61 95% CI: 0.54–0.66), CAR (AUC = 0. 67 95% CI: 0.62–0.73), RAR (AUC = 0.66 95% CI: 0.61–0.72), SOFA (AUC = 0.67 95% CI: 0.61–0.73), OASIS (AUC = 0.67 95% CI: 0.61–0.73). The AUC of BAR was lower than OASIS score for death within ICU 60 days [BAR (AUC = 0.75 95% CI: 0.70–0.81), OASIS (AUC = 0.76 95% CI: 0.70–0.83), Therefore, BAR has a significant advantage in predicting total mortality after ICU admission (S2 Table).The cumulative risk of deaths occurring in the BAR ≥ 7.62 group was significantly higher than BAR < 7.62 group (P < 0.05) at various time intervals (Fig 4).

### 3.5. Subgroup analyses of BAR at different periods

**Fig 5:**
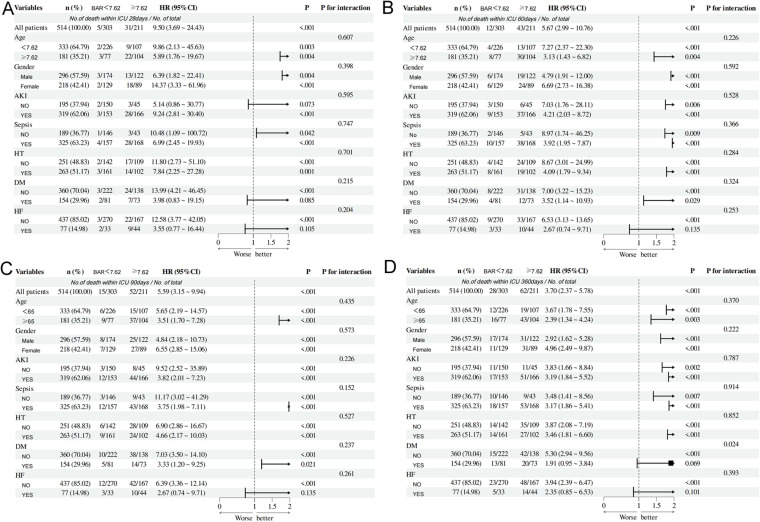
Subgroup analysis of AP patients in ICU at different time periods Forest plot illustrate the correlation between total mortality and BAR in different subgroups for AP patients admitted to ICU within (A)28 days, (B) 60 days, (C) 90 days, (D) 360 days.

Subgroup analyses were performed in order to exclude the heterogeneity caused by confounding factors such as age, gender, and comorbidity (Fig 5). In predicting 360-day all-cause mortality post ICU admission,BAR was influenced by diabetes(P for interaction = 0.024).In contrast, no interactions were identified between the BAR and all subgroups across 28, 60, and 90-days intervals.(P for interaction > 0.05) (S3 Table).The results indicate BAR serves as a stable independent predictor.

## 4. Discussion

In recent years, the predictive effect of serum markers on the prognosis of ICU patients is a major research hotspot, such as red blood cell distribution width to albumin ratio(RAR), lactate/albumin ratio (LAR), creatinine to albumin ratio (CAR), glucose to lymphocyte ratio (GLR) on the prognosis of AP, and the predictive effects of BAR on the prognosis of patients with sepsis, chronic obstructive pulmonary disease (COPD), and post-cardiac surgery [13–16]. However, the role of BAR in the relationship between death and survival of AP patients after ICU admission is still unknown. Our study provides conclusive evidence that BAR emerged as an independent predictor for overall mortality within 28, 60, 90 and 360 days after ICU admission. According to the cumulative hazard risk accumulation curves, patients with AP and BAR ≥ 7.62 had obviously superior mortality than low BAR at 28, 60, 90, and 360 days. Meanwhile, according to the AUC, the predictive effect of BAR on patients’ poor prognosis surpasses that of RAR, LAR, and CAR in existing studies, and also better than traditional SOFA and OASIS scores. By subgroup analysis, we excluded the impact of heterogeneity. BAR demonstrated a superior capacity to predict total death risk with no significant interaction with subgroups (P > 0.05). Previous studies indicate that BAR cutoff values serve as a predictor for various patient populations such as post-cardiac surgery, 6.41, Sepsis, 8.0, COVID-19,4.94, AECOPD (ALB unit g/L),0.249, and patients admitted to the surgical ICU,9.69 [7–11]. The values differ significantly, variations in cutoff values stem from disease mechanisms and inclusion criteria. However, BAR is an independent predictive metric that positively correlates with increased mortality. The validation of our research findings is also substantiated. The BAR cutoff value cannot be a universal indicator similar to assessing anemia based on hemoglobin levels, it can be customized to create individualized standards depending on specific disease conditions.

Studies have shown that ALB has a variety of biological effects, such as maintaining osmotic pressure, participating in drug binding and transport, scavenging free radicals, antioxidant, maintaining microvascular integrity, and thus reducing intravascular fluid leakage [17–20]. Hypoalbuminemia is a confirmed risk factor for poor prognosis in seriously ill patients [21]. A waterfall-like inflammatory response is present in the early stage of AP patients. The inflammatory response accelerates the process of protein hydrolysis, thereby reducing the levels of ALB, the plasma colloidal osmotic pressure correlates positively with ALB level. At the same time, a large amount of fluid leaks out of the blood vessels into the tissue interstitial space, resulting in insufficient effective volume of blood and reduced cardiac output, causing multiple organ hypoperfusion and activation of the renin-angiotensin-aldosterone system (RAAS) and the sympathetic nervous system [22]. Angiotensin and epinephrine stimulate renal vasoconstriction, reducing glomerular filtration rate (GFR) and renal blood flow, thereby enhancing urea reabsorption [23].

BUN is a nitrogen-containing compound with a small molecular weight, generated through the metabolic hydrolysis of proteins and excreted by the kidneys [24], which reflects absorption of protein, nutritional status, endogenous proteolytic metabolism, liquid balance, urea synthesis, and renal function of serious patients [25–27].

ALB and BUN are independent risk factors for many serious diseases. However, ALB levels are affected by age, calorie intake, inflammatory response, and chronic diseases, whereas BUN is influenced by protein intake, blood capacity, renal tubular reabsorption and urea excretion. The predictive value of a single variable for prognosis is significantly limited [28]. Existing studies show that BUN is less effective than BUN to ALB radio for predicting sepsis prognosis [7], while lactate to ALB ratio outperforms ALB in forecasting ICU patient outcomes [29]. Therefore, our study used BUN/ALB combined indicators to enhance prognostic predictive value.

Previous research indicate that concentration of BUN and ALB can be affected by renal function, sepsis, age, etc, which are more prominent in elderly patients [30–34]. We performed univariate COX regression analyses and found that BAR was an independent predictor of total mortality for AP patients. In the multivariate Cox regression models, we adjusted for confounders such as age, gender, temperature, complications, clinical scores, auxiliary examination, treatment intervention, and BAR remained strongly associated with poor prognosis. In subgroup analyses, the BAR ≥ 7.62 group still demonstrated a consistent effect with other subgroups for patients aged > 65 years with comorbid CKD. Current research evaluates the prognostic predictive ability of BAR, SOFA, and OASIS via AUC, revealing no consistent advantage in one direction [35–37]. Similarly, our research observed discontinuities in the one-sided advantages of BAR and OASIS. Perhaps the statistical errors from a retrospective study utilizing the MIMIC database, characterized by single-center and limited sample size, may explain why the AUC of BAR at 60 days does not exhibit the continuous linear relationship seen with OASIS at 28, 90, and 360 days. The statement above does not negate that BAR as an independent predictor. In addition, there was an interaction between AP patients with diabetes and BAR at 360 days after admission to ICU (P for interaction = 0.024), The final outcome may be biased due to sample volume limitations, particularly after grouping.

Based on research utilizing real-world datasets, the BAR demonstrates a significant correlation with mortality among ICU patients diagnosed with AP. However, this study is not without its limitations. Firstly, the current investigation constitutes a single-center retrospective cohort study, future research should use multi-center large sample data to validate the predictive reliability of BAR for AP patients or conduct multi-center prospective studies. Second, this study only analysed the data of the first admission to ICU and did not dynamically assess the impact of BAR values at different time points on clinical outcomes of AP patients. Third, the MIMIC-IV database provides data on inpatients from 2008 to 2019, with a long time span. Considering the updates and changes in medical monitoring technology and disease treatment methods, the organ damage observed in patients with AP varies significantly among individuals. The implementation of individualized treatment plans, coupled with inconsistent baseline characteristics, will inevitably influence the outcomes. Fourth, the database includes both ICD-9 and ICD-10 codes. Unlike ICD-10, ICD-9 lacks detailed etiology diagnoses, restricting subgroup analyses for AP based on etiology.Finally, this study is a retrospective analysis of public databases, and patients with a high number of missing values of some indicators have not been included for analysis, so selection bias and confounding bias is inevitable.

## 5. Conclusions

BAR is an objective and independent predictor of both short- and long-term total mortality in AP patients. A prompt, efficient, and uncomplicated assessment of the severity and prognosis, which facilitates ICU doctors to develop treatment plans for poor patient outcomes.

## Supporting information

S1 Table Univariate COX analysis for expired within 28 days after AP admission. (DOCX)

S2 Table ROC for total mortality in AP were analysed and compared with BAR, RAR, CAR, LAR, SOFA and OASIS scores in 28, 60, 90, and 360days. (DOCX)

S3 Table Subgroup analysis of AP in ICU at different time periods(28, 60, 90, and 360days). (DOCX)

S4 Raw data. (CSV)
