# Supplementary material for: Blood urea nitrogen to albumin ratio as predictor of mortality among acute pancreatitis patients in ICU: A retrospective cohort study
Source: PLoS One. 2025 May 15;20(5):e0323321. doi: 10.1371/journal.pone.0323321 (PMC12080803; doi:10.1371/journal.pone.0323321)
Supplement: S1 Table — (DOCX) [file pone.0323321.s001.docx]

**S1 Table.** Univariate COX analysis for expired within 28 days after AP admission

| **Variables** | **N** | **HR** | **95%CI** | **P-value** |
| --- | --- | --- | --- | --- |
| Demographics | | | | |
| Age | 514.0 | 1.06 | [1.04,1.09] | 0.00 |
| Gender |  |  |  |  |
| Male | 296.0 |  |  |  |
| Female | 218.0 | 1.75 | [0.91,3.38] | 0.09 |
| Weight | 514.0 | 0.99 | [0.98,1.01] | 0.20 |
| Vital Signs | | | | |
| HR | 514.0 | 1.00 | [0.99,1.02] | 0.63 |
| RR | 514.0 | 1.00 | [0.95,1.04] | 0.84 |
| SPO2 | 514.0 | 1.03 | [0.94,1.13] | 0.57 |
| Nbpm | 514.0 | 0.99 | [0.98,1.01] | 0.38 |
| T | 514.0 | 0.58 | [0.44,0.77] | 0.00 |
| Laboratory data | | | | |
| WBC | 514.0 | 1.02 | [0.99,1.06] | 0.26 |
| RBC | 514.0 | 1.01 | [0.68,1.50] | 0.97 |
| Plt | 514.0 | 1.00 | [1.00,1.00] | 0.62 |
| Hb | 514.0 | 0.95 | [0.83,1.10] | 0.50 |
| RDW | 514.0 | 1.26 | [1.09,1.46] | 0.00 |
| Hct | 514.0 | 1.00 | [0.95,1.05] | 0.92 |
| Alb | 514.0 | 0.25 | [0.14,0.44] | 0.00 |
| TBil | 514.0 | 1.03 | [0.93,1.15] | 0.56 |
| ALT | 514.0 | 1.00 | [1.00,1.00] | 0.94 |
| AST | 514.0 | 1.00 | [1.00,1.00] | 0.94 |
| BUN | 514.0 | 1.02 | [1.01,1.02] | 0.00 |
| Cr | 514.0 | 1.14 | [1.01,1.29] | 0.04 |
| Na | 514.0 | 1.09 | [1.04,1.14] | 0.00 |
| K | 514.0 | 1.25 | [0.91,1.72] | 0.17 |
| TCa2 | 514.0 | 0.87 | [0.67,1.13] | 0.28 |
| Cl | 514.0 | 1.07 | [1.03,1.12] | 0.00 |
| Glu | 514.0 | 1.00 | [1.00,1.00] | 0.51 |
| PH | 514.0 | 0.06 | [0.00,0.93] | 0.04 |
| Lac | 514.0 | 1.18 | [1.08,1.30] | 0.00 |
| AG | 514.0 | 1.07 | [1.02,1.13] | 0.01 |
| PT | 514.0 | 1.01 | [0.98,1.06] | 0.46 |
| APTT | 514.0 | 1.01 | [1.00,1.03] | 0.09 |
| INR | 514.0 | 1.17 | [0.81,1.70] | 0.40 |
| BAR |  |  |  |  |
| ＜7.62 | 303.0 |  |  |  |
| ≥7.62 | 211.0 | 9.50 | [3.69,24.43] | 0.00 |
| Scores | | | | |
| SOFA | 514.0 | 1.22 | [1.13,1.31] | 0.00 |
| OASIS | 514.0 | 1.10 | [1.06,1.13] | 0.00 |
| Comorbidities | | | | |
| AKI |  |  |  |  |
| NO | 195.0 |  |  |  |
| YES | 319.0 | 3.93 | [1.53,10.10] | 0.00 |
| Sepsis |  |  |  |  |
| NO | 189.0 |  |  |  |
| YES | 325.0 | 4.85 | [1.71,13.71] | 0.00 |
| HT |  |  |  |  |
| NO | 251.0 |  |  |  |
| YES | 263.0 | 0.86 | [0.45,1.66] | 0.66 |
| DM |  |  |  |  |
| NO | 360.0 |  |  |  |
| YES | 154.0 | 0.77 | [0.36,1.65] | 0.51 |
| HF |  |  |  |  |
| NO | 437.0 |  |  |  |
| YES | 77.0 | 2.58 | [1.27,5.25] | 0.01 |
| CKD |  |  |  |  |
| NO | 457.0 |  |  |  |
| YES | 57.0 | 2.34 | [1.07,5.13] | 0.03 |
| Clinical treatments | | | | |
| MV |  |  |  |  |
| NO | 311.0 |  |  |  |
| YES | 203.0 | 3.17 | [1.59,6.34] | 0.00 |
| Norepinephrine |  |  |  |  |
| NO | 383.0 |  |  |  |
| YES | 131.0 | 7.28 | [3.58,14.80] | 0.00 |
| Vasopressin |  |  |  |  |
| NO | 449.0 |  |  |  |
| YES | 65.0 | 8.07 | [4.20,15.52] | 0.00 |
