# Supplementary material for: Blood urea nitrogen to albumin ratio as predictor of mortality among acute pancreatitis patients in ICU: A retrospective cohort study
Source: PLoS One. 2025 May 15;20(5):e0323321. doi: 10.1371/journal.pone.0323321 (PMC12080803; doi:10.1371/journal.pone.0323321)
Supplement: S2 Table — (DOCX) [file pone.0323321.s002.docx]

**S2 Table.** ROC for total mortality in AP were analysed and compared with BAR, RAR, CAR, LAR, SOFA and OASIS scores in 28, 60, 90, and 360days

| **Variable** | **AUC** | **Sensitivity** | **Specificity** | **Youden Index** | **Cut-off Value** |
| --- | --- | --- | --- | --- | --- |
| 28days | | | | | |
| BAR | 0.78(0.72-0.84) | 0.86(0.68-1.00) | 0.62(0.47-0.80) | 0.48(0.39-0.60) | 7.62(5.71-11.97) |
| LAR | 0.70(0.61-0.78) | 0.78(0.42-0.94) | 0.53(0.35-0.90) | 0.30(0.23-0.47) | 0.61(0.51-1.40) |
| CAR | 0.73(0.65-0.80) | 0.89(0.56-0.97) | 0.49(0.43-0.85) | 0.38(0.28-0.53) | 0.32(0.29-0.76) |
| RAR | 0.76(0.66-0.85) | 0.81(0.54-0.93) | 0.66(0.62-0.88) | 0.46(0.36-0.59) | 5.54(5.54-6.55) |
| SOFA | 0.74(0.67-0.82) | 0.58(0.44-0.94) | 0.76(0.40-0.85) | 0.34(0.26-0.53) | 8.00(4.00-9.52) |
| OASIS | 0.78(0.69-0.84) | 0.67(0.59-0.93) | 0.77(0.55-0.83) | 0.44(0.36-0.59) | 39.00(33.00-40.00) |
| 60days | | | | | |
| BAR | 0.75(0.69-0.80) | 0.80(0.73-1.00) | 0.63(0.39-0.70) | 0.43(0.36-0.54) | 7.58(4.69-8.91) |
| LAR | 0.69(0.64-0.76) | 0.49(0.39-0.97) | 0.79(0.34-0.88) | 0.28(0.23-0.42) | 1.00(0.47-1.34) |
| CAR | 0.71(0.64-0.77) | 0.84(0.58-0.94) | 0.50(0.40-0.77) | 0.34(0.26-0.48) | 0.32(0.28-0.55) |
| RAR | 0.74(0.66-0.80) | 0.75(0.54-0.85) | 0.67(0.64-0.88) | 0.41(0.32-0.54) | 5.54(5.54-6.52) |
| SOFA | 0.73(0.67-0.80) | 0.58(0.53-0.95) | 0.78(0.38-0.82) | 0.36(0.27-0.52) | 8.00(4.00-9.00) |
| OASIS | 0.76(0.69-0.82) | 0.60(0.56-0.96) | 0.81(0.46-0.83) | 0.41(0.37-0.52) | 40.00(31.00-40.00) |
| 90days | | | | | |
| BAR | 0.75(0.71-0.80) | 0.79(0.72-0.98) | 0.64(0.38-0.71) | 0.43(0.34-0.53) | 7.58(4.69-9.20) |
| LAR | 0.66(0.60-0.73) | 0.76(0.33-0.91) | 0.48(0.35-0.90) | 0.24(0.20-0.37) | 0.57(0.51-1.38) |
| CAR | 0.70(0.64-0.77) | 0.63(0.58-0.94) | 0.70(0.37-0.74) | 0.32(0.25-0.47) | 0.47(0.26-0.49) |
| RAR | 0.70(0.62-0.77) | 0.49(0.41-0.73) | 0.87(0.66-0.90) | 0.36(0.26-0.49) | 6.48(5.54-6.52) |
| SOFA | 0.71(0.65-0.77) | 0.54(0.45-0.92) | 0.78(0.40-0.83) | 0.32(0.23-0.45) | 8.00(4.00-9.00) |
| OASIS | 0.73(0.67-0.80) | 0.55(0.48-0.92) | 0.81(0.45-0.85) | 0.37(0.29-0.52) | 40.00(31.00-40.00) |
| 360days | | | | | |
| BAR | 0.70(0.64-0.75) | 0.70(0.61-0.91) | 0.65(0.37-0.71) | 0.35(0.26-0.45) | 7.58(4.57-8.91) |
| LAR | 0.61(0.53-0.67) | 0.38(0.17-0.91) | 0.79(0.28-0.97) | 0.17(0.11-0.30) | 1.00(0.44-1.86) |
| CAR | 0.67(0.61-0.73) | 0.82(0.45-0.94) | 0.45(0.32-0.85) | 0.27(0.20-0.40) | 0.29(0.25-0.80) |
| RAR | 0.66(0.60-0.72) | 0.41(0.31-0.72) | 0.87(0.58-0.95) | 0.28(0.21-0.41) | 6.48(5.15-7.60) |
| SOFA | 0.67(0.61-0.72) | 0.63(0.47-0.89) | 0.61(0.37-0.79) | 0.24(0.17-0.35) | 6.00(4.00-8.00) |
| OASIS | 0.67(0.62-0.73) | 0.47(0.42-0.84) | 0.82(0.46-0.85) | 0.28(0.21-0.39) | 40.00(30.48-40.00) |
