# Supplementary material for: Blood urea nitrogen to albumin ratio as predictor of mortality among acute pancreatitis patients in ICU: A retrospective cohort study
Source: PLoS One. 2025 May 15;20(5):e0323321. doi: 10.1371/journal.pone.0323321 (PMC12080803; doi:10.1371/journal.pone.0323321)
Supplement: S3 Table — (DOCX) [file pone.0323321.s003.docx]

### **S3 Table.** Subgroup analysis of AP in ICU at different time periods(28, 60, 90, and 360days)

**28days**

| Variables | n (%) | BAR＜7.62 | BAR≥7.62 | HR (95%CI) | *P* | P for interaction |
| --- | --- | --- | --- | --- | --- | --- |
|  |  |  |  |  |  |  |
| All patients | 514 (100.00) | 5/303 | 31/211 | 9.50 (3.69 ~ 24.43) | **<.001** |  |
| Age |  |  |  |  |  | 0.607 |
| ＜65 | 333 (64.79) | 2/226 | 9/107 | 9.86 (2.13 ~ 45.63) | **0.003** |  |
| ≥65 | 181 (35.21) | 3/77 | 22/104 | 5.89 (1.76 ~ 19.67) | **0.004** |  |
| Gender |  |  |  |  |  | 0.398 |
| Male | 296 (57.59) | 3/174 | 13/122 | 6.39 (1.82 ~ 22.41) | **0.004** |  |
| Female | 218 (42.41) | 2/129 | 18/89 | 14.37 (3.33 ~ 61.96) | **<.001** |  |
| AKI |  |  |  |  |  | 0.595 |
| NO | 195 (37.94) | 2/150 | 3/45 | 5.14 (0.86 ~ 30.77) | 0.073 |  |
| YES | 319 (62.06) | 3/153 | 28/166 | 9.24 (2.81 ~ 30.40) | **<.001** |  |
| Sepsis |  |  |  |  |  | 0.747 |
| NO | 189 (36.77) | 1/146 | 3/43 | 10.48 (1.09 ~ 100.72) | **0.042** |  |
| YES | 325 (63.23) | 4/157 | 28/168 | 6.99 (2.45 ~ 19.93) | **<.001** |  |
| HT |  |  |  |  |  | 0.701 |
| NO | 251 (48.83) | 2/142 | 17/109 | 11.80 (2.73 ~ 51.10) | **<.001** |  |
| YES | 263 (51.17) | 3/161 | 14/102 | 7.84 (2.25 ~ 27.28) | **0.001** |  |
| DM |  |  |  |  |  | 0.215 |
| NO | 360 (70.04) | 3/222 | 24/138 | 13.99 (4.21 ~ 46.45) | **<.001** |  |
| YES | 154 (29.96) | 2/81 | 7/73 | 3.98 (0.83 ~ 19.15) | 0.085 |  |
| HF |  |  |  |  |  | 0.204 |
| NO | 437 (85.02) | 3/270 | 22/167 | 12.58 (3.77 ~ 42.05) | **<.001** |  |
| YES | 77 (14.98) | 2/33 | 9/44 | 3.55 (0.77 ~ 16.44) | 0.105 |  |
| CKD |  |  |  |  |  | 0.164 |
| NO | 457 (88.91) | 4/290 | 24/167 | 11.12 (3.86 ~ 32.06) | **<.001** |  |
| YES | 57 (11.09) | 1/13 | 7/44 | 2.11 (0.26 ~ 17.13) | 0.486 |  |
| HR: Hazard Ratio, CI: Confidence Interval  BAR, Serum urea nitrogen to albumin ratio; AKI, acute kidney injury; HT, Hypertension; DM, diabetes; HF, heart failure; CKD, chronic kidney disease. | | | | | | |

**60days**

| Variables | n (%) | BAR＜7.62 | BAR≥7.62 | HR (95%CI) | *P* | P for interaction |
| --- | --- | --- | --- | --- | --- | --- |
|  |  |  |  |  |  |  |
| All patients | 514 (100.00) | 12/303 | 43/211 | 5.67 (2.99 ~ 10.76) | **<.001** |  |
| Age |  |  |  |  |  | 0.226 |
| ＜65 | 333 (64.79) | 4/226 | 13/107 | 7.27 (2.37 ~ 22.30) | **<.001** |  |
| ≥65 | 181 (35.21) | 8/77 | 30/104 | 3.13 (1.43 ~ 6.82) | **0.004** |  |
| Gender |  |  |  |  |  | 0.592 |
| Male | 296 (57.59) | 6/174 | 19/122 | 4.79 (1.91 ~ 12.00) | **<.001** |  |
| Female | 218 (42.41) | 6/129 | 24/89 | 6.69 (2.73 ~ 16.38) | **<.001** |  |
| AKI |  |  |  |  |  | 0.528 |
| NO | 195 (37.94) | 3/150 | 6/45 | 7.03 (1.76 ~ 28.11) | **0.006** |  |
| YES | 319 (62.06) | 9/153 | 37/166 | 4.21 (2.03 ~ 8.72) | **<.001** |  |
| Sepsis |  |  |  |  |  | 0.366 |
| NO | 189 (36.77) | 2/146 | 5/43 | 8.97 (1.74 ~ 46.25) | **0.009** |  |
| YES | 325 (63.23) | 10/157 | 38/168 | 3.92 (1.95 ~ 7.87) | **<.001** |  |
| HT |  |  |  |  |  | 0.284 |
| NO | 251 (48.83) | 4/142 | 24/109 | 8.67 (3.01 ~ 24.99) | **<.001** |  |
| YES | 263 (51.17) | 8/161 | 19/102 | 4.09 (1.79 ~ 9.34) | **<.001** |  |
| DM |  |  |  |  |  | 0.324 |
| NO | 360 (70.04) | 8/222 | 31/138 | 7.00 (3.22 ~ 15.23) | **<.001** |  |
| YES | 154 (29.96) | 4/81 | 12/73 | 3.52 (1.14 ~ 10.93) | **0.029** |  |
| HF |  |  |  |  |  | 0.253 |
| NO | 437 (85.02) | 9/270 | 33/167 | 6.53 (3.13 ~ 13.65) | **<.001** |  |
| YES | 77 (14.98) | 3/33 | 10/44 | 2.67 (0.74 ~ 9.71) | 0.135 |  |
| CKD |  |  |  |  |  | 0.663 |
| NO | 457 (88.91) | 11/290 | 32/167 | 5.56 (2.80 ~ 11.03) | **<.001** |  |
| YES | 57 (11.09) | 1/13 | 11/44 | 3.45 (0.45 ~ 26.71) | 0.236 |  |
| HR: Hazard Ratio, CI: Confidence Interval  BAR, Serum urea nitrogen to albumin ratio; AKI, acute kidney injury; HT, Hypertension; DM, diabetes; HF, heart failure; CKD, chronic kidney disease. | | | | | | |

**90days**

| Variables | n (%) | BAR＜7.62 | BAR≥7.62 | HR (95%CI) | *P* | P for interaction |
| --- | --- | --- | --- | --- | --- | --- |
|  |  |  |  |  |  |  |
| All patients | 514 (100.00) | 15/303 | 52/211 | 5.59 (3.15 ~ 9.94) | **<.001** |  |
| Age |  |  |  |  |  | 0.435 |
| ＜65 | 333 (64.79) | 6/226 | 15/107 | 5.65 (2.19 ~ 14.57) | **<.001** |  |
| ≥65 | 181 (35.21) | 9/77 | 37/104 | 3.51 (1.70 ~ 7.28) | **<.001** |  |
| Gender |  |  |  |  |  | 0.573 |
| Male | 296 (57.59) | 8/174 | 25/122 | 4.84 (2.18 ~ 10.73) | **<.001** |  |
| Female | 218 (42.41) | 7/129 | 27/89 | 6.55 (2.85 ~ 15.06) | **<.001** |  |
| AKI |  |  |  |  |  | 0.226 |
| NO | 195 (37.94) | 3/150 | 8/45 | 9.52 (2.52 ~ 35.89) | **<.001** |  |
| YES | 319 (62.06) | 12/153 | 44/166 | 3.82 (2.01 ~ 7.23) | **<.001** |  |
| Sepsis |  |  |  |  |  | 0.152 |
| NO | 189 (36.77) | 3/146 | 9/43 | 11.17 (3.02 ~ 41.29) | **<.001** |  |
| YES | 325 (63.23) | 12/157 | 43/168 | 3.75 (1.98 ~ 7.11) | **<.001** |  |
| HT |  |  |  |  |  | 0.527 |
| NO | 251 (48.83) | 6/142 | 28/109 | 6.90 (2.86 ~ 16.67) | **<.001** |  |
| YES | 263 (51.17) | 9/161 | 24/102 | 4.66 (2.17 ~ 10.03) | **<.001** |  |
| DM |  |  |  |  |  | 0.237 |
| NO | 360 (70.04) | 10/222 | 38/138 | 7.03 (3.50 ~ 14.10) | **<.001** |  |
| YES | 154 (29.96) | 5/81 | 14/73 | 3.33 (1.20 ~ 9.25) | **0.021** |  |
| HF |  |  |  |  |  | 0.261 |
| NO | 437 (85.02) | 12/270 | 42/167 | 6.39 (3.36 ~ 12.14) | **<.001** |  |
| YES | 77 (14.98) | 3/33 | 10/44 | 2.67 (0.74 ~ 9.71) | 0.135 |  |
| CKD |  |  |  |  |  | 0.817 |
| NO | 457 (88.91) | 14/290 | 39/167 | 5.41 (2.94 ~ 9.97) | **<.001** |  |
| YES | 57 (11.09) | 1/13 | 13/44 | 4.18 (0.55 ~ 31.95) | 0.168 |  |
| HR: Hazard Ratio, CI: Confidence Interval  BAR, Serum urea nitrogen to albumin ratio; AKI, acute kidney injury; HT, Hypertension; DM, diabetes; HF, heart failure; CKD, chronic kidney disease. | | | | | | |

**360days**

| Variables | n (%) | BAR＜7.62 | BAR≥7.62 | HR (95%CI) | *P* | P for interaction |
| --- | --- | --- | --- | --- | --- | --- |
|  |  |  |  |  |  |  |
| All patients | 514 (100.00) | 28/303 | 62/211 | 3.70 (2.37 ~ 5.78) | **<.001** |  |
| Age |  |  |  |  |  | 0.370 |
| ＜65 | 333 (64.79) | 12/226 | 19/107 | 3.67 (1.78 ~ 7.55) | **<.001** |  |
| ≥65 | 181 (35.21) | 16/77 | 43/104 | 2.39 (1.34 ~ 4.24) | **0.003** |  |
| Gender |  |  |  |  |  | 0.222 |
| Male | 296 (57.59) | 17/174 | 31/122 | 2.92 (1.62 ~ 5.28) | **<.001** |  |
| Female | 218 (42.41) | 11/129 | 31/89 | 4.96 (2.49 ~ 9.87) | **<.001** |  |
| AKI |  |  |  |  |  | 0.787 |
| NO | 195 (37.94) | 11/150 | 11/45 | 3.83 (1.66 ~ 8.84) | **0.002** |  |
| YES | 319 (62.06) | 17/153 | 51/166 | 3.19 (1.84 ~ 5.52) | **<.001** |  |
| Sepsis |  |  |  |  |  | 0.914 |
| NO | 189 (36.77) | 10/146 | 9/43 | 3.48 (1.41 ~ 8.56) | **0.007** |  |
| YES | 325 (63.23) | 18/157 | 53/168 | 3.17 (1.86 ~ 5.41) | **<.001** |  |
| HT |  |  |  |  |  | 0.852 |
| NO | 251 (48.83) | 14/142 | 35/109 | 3.87 (2.08 ~ 7.19) | **<.001** |  |
| YES | 263 (51.17) | 14/161 | 27/102 | 3.46 (1.81 ~ 6.60) | **<.001** |  |
| DM |  |  |  |  |  | 0.024 |
| NO | 360 (70.04) | 15/222 | 42/138 | 5.30 (2.94 ~ 9.56) | **<.001** |  |
| YES | 154 (29.96) | 13/81 | 20/73 | 1.91 (0.95 ~ 3.84) | 0.069 |  |
| HF |  |  |  |  |  | 0.393 |
| NO | 437 (85.02) | 23/270 | 48/167 | 3.94 (2.39 ~ 6.47) | **<.001** |  |
| YES | 77 (14.98) | 5/33 | 14/44 | 2.35 (0.85 ~ 6.53) | 0.101 |  |
| CKD |  |  |  |  |  | 0.648 |
| NO | 457 (88.91) | 27/290 | 46/167 | 3.42 (2.12 ~ 5.49) | **<.001** |  |
| YES | 57 (11.09) | 1/13 | 16/44 | 5.36 (0.71 ~ 40.47) | 0.103 |  |
| HR: Hazard Ratio, CI: Confidence Interval  BAR, Serum urea nitrogen to albumin ratio; AKI, acute kidney injury; HT, Hypertension; DM, diabetes; HF, heart failure; CKD, chronic kidney disease. | | | | | | |
